# Re-evaluation of the near infrared spectra of mitochondrial cytochrome *c* oxidase: Implications for non invasive in vivo monitoring of tissues

**DOI:** 10.1016/j.bbabio.2014.08.005

**Published:** 2014-11

**Authors:** Maria G. Mason, Peter Nicholls, Chris E. Cooper

**Affiliations:** School of Biological Sciences, University of Essex, Wivenhoe Park, Colchester CO4 3SQ, UK

**Keywords:** TMPD, N,N,N′,N′-Tetramethyl-p-phenylenediamine, NIR, near infrared, Near infrared spectroscopy, NIR, Mitochondria, Cytochrome oxidase, Copper, Cu_A_

## Abstract

We re-determined the near infrared (NIR) spectral signatures (650–980 nm) of the different cytochrome *c* oxidase redox centres, in the process separating them into their component species. We confirm that the primary contributor to the oxidase NIR spectrum between 700 and 980 nm is cupric Cu_A_, which in the beef heart enzyme has a maximum at 835 nm. The 655 nm band characterises the fully oxidised haem *a*_3_/Cu_B_ binuclear centre; it is bleached either when one or more electrons are added to the binuclear centre or when the latter is modified by ligands. The resulting ‘perturbed’ binuclear centre is also characterised by a previously unreported broad 715–920 nm band. The NIR spectra of certain stable liganded species (formate and CO), and the unstable oxygen reaction compounds P and F, are similar, suggesting that the latter may resemble the stable species electronically. Oxidoreduction of haem *a* makes no contribution either to the 835 nm maximum or the 715 nm band. Our results confirm the ability of NIRS to monitor the Cu_A_ centre of cytochrome oxidase activity in vivo, although noting some difficulties in precise quantitative interpretations in the presence of perturbations of the haem *a*_3_/Cu_B_ binuclear centre.

## Highlights

• The NIR spectrum of cytochrome oxidase was deconvoluted into its component species.
• The dominant feature between 700 and 980 nm was confirmed as the CuA chromophore.
• There was no significant contribution from the haem *a* iron centre.
• A new feature between 715 and 920 nm was assigned to the haem *a*_3_/Cu_B_ binuclear centre.
• Changes in concentrations of oxygen intermediates P and F may be measurable in vivo.

## Graphical abstract

**Figure:**
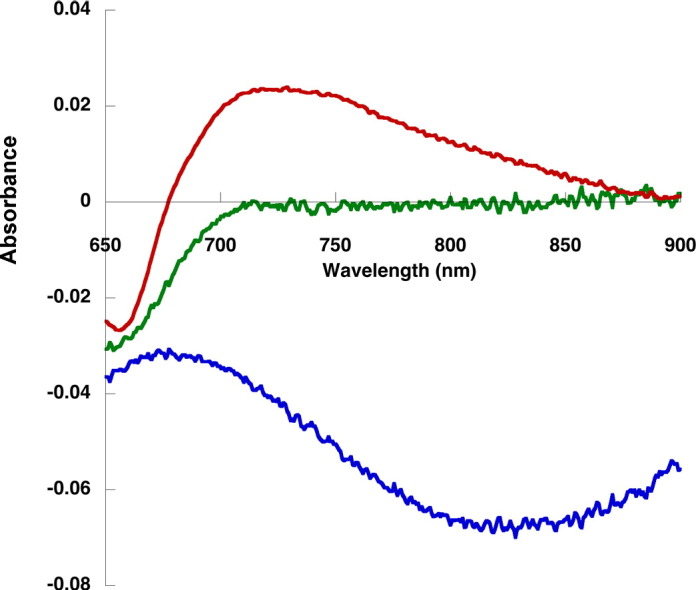

## 1 Introduction

Mitochondrial cytochrome *c* oxidase is now known to contain four redox active metal centres, two haem and two copper. Historically optical spectroscopy focussed on the strong visible light signals from the haem iron centres, cytochrome *a* [1] and *a*_3_ [2]. Any copper was regarded as adventitious [3]. This view changed when electron paramagnetic resonance (EPR) spectroscopy showed detectable copper signals. However, only part of the copper, the ‘visible’ form (now termed Cu_A_), was detectable; this cupric signal tracked enzyme turnover [4], [5] and was linked to parallel near infrared (NIR) optical changes at 830 nm [6].

Chance followed NIR changes in submitochondrial particles but found slow redox behaviour [7]. In contrast, the first detailed kinetic studies by Greenwood and Gibson showed that nearly all the 835 nm component appeared rapidly on pulsing isolated reduced enzyme with oxygen [8], [9]. Existence of a second ‘invisible’ copper centre (now termed Cu_B_), complicated the analysis. Van Gelder claimed one copper centre was responsible for half the NIR absorbance [10], [11]. Despite the introduction of techniques for probing EPR invisible copper, such as EXAFS, there remained doubts as to the relative contributions of haems and coppers to the NIR absorbance [12]. However, persuasive evidence for Cu_A_ being the dominant NIR chromophore came when it was shown that strong NIR signals were obtained from isolated protein subunits containing only this chromophore [13]. This view was confirmed when it was shown that homologous bacterial enzymes that lack the Cu_A_ centre also lack the corresponding strong NIR absorbance [14].

However, several unresolved questions remain: Cu_B_ reduction perturbs the 655 nm band in the oxidised enzyme [15], [16]; haem *a* spectral shifts occur upon inhibitory ligand binding to the binuclear centre [17]; the reduced *a*_3_Cu_B_ spectrum may include Cu_B_-dependent as well as haem *a*_3_ components [18], [19], [20]; and other minor NIR absorbance bands are present, generally attributed to ferrous haem *a*_3_ [21], [22], [23]. Distinguishing different NIR spectral changes is thus essential to analysing oxidase function in vitro.

The NIR ‘window’ from 680 to 980 nm is also key to monitoring the enzyme in mitochondria, intact cells and whole organisms [24], [25] and so to linking enzymatic events with global oxygen metabolism in vivo [26]. Work with intact mammals requires special arrangements to remove the spectroscopic contributions from haemoglobin. Full cytochrome spectroscopy can sometimes be achieved as with bloodless rats, [27], [28], but in human beings or other large animals only the NIR ‘window’ can be used [29].

Discussion has been on-going for a number of years as to whether oxidase activity and control are identical in vitro and in vivo or whether the in vivo system shows unique features. Classical observations in this area were made by Jöbsis [30]. Unique in vivo steady state reduction patterns of cytochromes *a* and *a*_3_ were reported for cells and tissues. Current measurements with whole cells have also identified some unusual oxidase species [31]. The possibility of in vivo controls not readily seen in vitro was also emphasized more recently by the observation of labile enzyme phosphorylation in intact systems; unlike enzyme in situ the isolated mammalian enzyme is usually completely dephosphorylated [32].

A range of studies has shown that the NIR spectrum of cytochrome oxidase has potential as a monitor of brain oxygen delivery and/or metabolism [33], [34], [35], [36]. Therefore allocating the species contributing to the NIR absorbance changes has clinical as well as basic science relevance. Is the 830 nm signal seen in vivo solely due to cytochrome *c* oxidase cupric Cu_A_ or are there other contributors? If the signal is indeed due only to cupric Cu_A_ what does it tell us about the overall redox status of the enzyme? Does the redox state of Cu_A_ correlate well with oxidase turnover? Our most recent in vitro results [16], [37] were interpreted in terms of Cu_A_ being always in near equilibrium with the enzyme's redox substrate, cytochrome *c* (although see Hoshi et al. [38] for an alternative view). Therefore as Cu_A_ is close to cytochrome *c* in redox potential, and the equilibrium between the two is minimally affected by the energy state of the mitochondrial inner membrane [39], the Cu_A_ redox state should reflect that of cytochrome *c*. In turn the latter is believed to provide a measure of oxidase activity.

We have therefore extended our previous analysis [16], [37] by examining all the possible NIR signal contributions from cytochrome *c* oxidase, focussing upon the changes in the NIR spectral region at high enzyme concentrations. We conclude, with some caveats, that the 830 nm band is indeed largely due to cupric Cu_A_ and that this monitors the enzyme turnover, as previously suggested. The NIR region can also provide information about the oxidase O_2_ reaction intermediates.

## 2 Materials and methods

### 2.1 Materials

Beef heart oxidase was prepared as described below. Cytochrome *c* was Sigma type VI (or equivalent) horse heart protein. Beef liver catalase was obtained by diluting Roche Diagnostics catalase 108 810 from beef liver in a slightly alkaline medium. A stock H_2_O_2_ sample was prepared from a 30% Sigma-Aldrich aqueous solution. Sodium dithionite, (+)-sodium ascorbate, sodium formate, N,N,N′,N′-tetramethyl-*p*-phenylenediamine dihydrochloride (TMPD) lauryl (dodecyl) maltoside, potassium ferricyanide and all buffer salts were analytical reagent or equivalent samples from Sigma-Aldrich. Sodium dithionite solutions (10% w/v) were prepared as required and used within 3 h. CO (carbon monoxide) was 100% BOC compressed gas.

### 2.2 Preparation of cytochrome *c* oxidase

The beef heart enzyme was prepared by the method of Yonetani [18] with minor modifications. Lauryl maltoside was employed as the final detergent and the detergent extraction was performed at 5 °C. The preparation had an absorbance ratio (280 nm/Soret) of ~ 2.4 with not more than 5% haem impurity (from analytical HPLC). It was assayed with a turnover number (reducing equivalents per mole cytochrome *aa*_3_ per second) of ~ 250 e^−^ s^− 1^ in the presence of ascorbate/cytochrome *c*/TMPD (10 mM/30 μM/300 μM) at pH 7.4 and at 35 °C.

### 2.3 Preparation of pulsed (fast) form of oxidised enzyme

The resting enzyme (3.6 ml of 75 μM stock cytochrome *aa*_3_), in the presence of a small amount of beef liver catalase, was reduced by the addition of freshly prepared 10% dithionite solution in 0.1 M Na Borate buffer pH 8.75 containing 170 nM catalase. The samples were incubated at room temperature for 15 min and a further 1 h on ice. The enzyme was diluted with buffer (0.1 M Na Borate/0.1% LM, pH 8.75) and then concentrated in a Vivaspin 20 (30,000 MW cut-off filter, Vivascience) by centrifugation (1 h, 1500 *g*); this process also removes low molecular weight contaminants produced by the treatment. To ensure complete re-oxidation of cytochrome oxidase, potassium ferricyanide aliquots and a further 6 ml buffer were added to the concentrated enzyme sample. Excess ferricyanide was removed by re-centrifugation in the Vivaspin device for a further 40 min. The ferricyanide treatment was repeated (once or twice) until the Soret and visible peak maxima at 423 and 598 nm in the optical spectrum were unchanging at pH 8.75. These pulsed enzyme samples (hereafter referred to as oxidised enzyme) were stored in the pH 8.75 buffer at − 80 °C until required.

### 2.4 Spectral acquisition

Data were acquired using Spectrasuite software and a fibre optic system comprising a cooled CCD (QE65000, 25 μm slit width), a tungsten halogen light source (HL-2000-FHSA-HP), 1000 μm optical fibres (QP1000-2-VIS-BX) and either a standard cuvette holder (CUV) (all from Ocean Optics) or a light-integrating sphere with centre mounted cuvette holder (Labsphere, Prolite). Unless otherwise stated the oxidised spectrum was recorded and stored with subsequent spectra acquired as difference spectra relative to oxidised.

### 2.5 Preparation of oxygen intermediates P & F

**Fig. 1:**
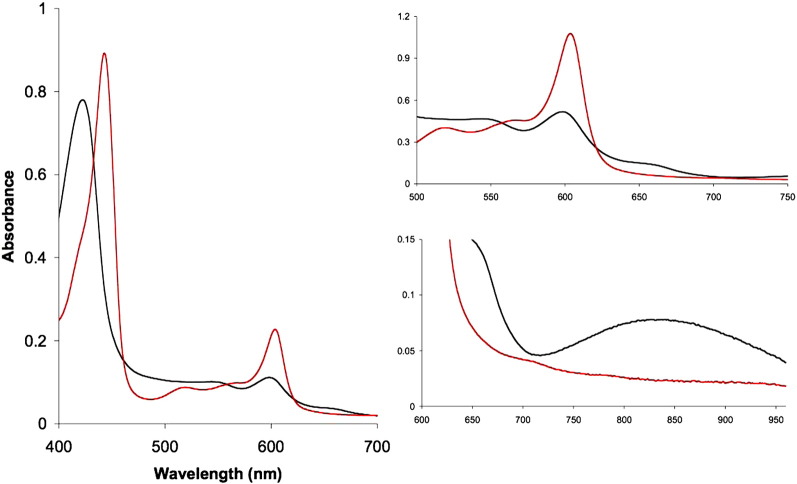
Optical features of oxidised & reduced cytochrome oxidase. Absolute spectra oxidised (black) and fully reduced (red), obtained in the Soret, visible and NIR regions. 0.1 M Na borate buffer pH 8.75 0.1% lauryl maltoside at 22°°C. Reduction achieved by addition of a small aliquot of sodium dithionite and acquiring the spectrum shown after 5 min. The main panel shows the spectrum of 4.9 μM enzyme, the inset spectra were acquired at a fivefold higher concentration. The oxidised enzyme spectrum has Soret and visible maxima at 423 nm and 598 nm, respectively. Derived extinction coefficients are shown in Table 1.

The P intermediate was obtained by gently blowing CO gas onto the enzyme for 10 s at pH 8.75, with subsequent incubation leading to complete formation after a few minutes. The amount of P formed was calculated using a delta E_M_ 607–630 nm = 11,000 M^− 1^ cm^− 1^ [40]. The F intermediate was obtained by the addition of peroxide at pH 8.75 (a small aliquot of catalase added during the pulsing procedure was not removed). Upon addition of 5 mM H_2_O_2_ a mixture of F & P was formed. The amounts of P and F involved were calculated using simultaneous equations with the extinction coefficients shown in Table 1. Fig. 1 shows the absolute spectra of the enzyme (reduced and oxidised) obtained with the QE65000 system (insets show the visible and NIR spectra on expanded scales). The derived extinction coefficients employed are summarised in Table 1. The absolute spectra of pulsed enzyme in the near infrared (NIR) region in both clear and scattering media were also obtained with the integrating sphere technique (results not shown). The spectra were essentially identical with those obtained by conventional spectrophotometry, indicating that none of the broad peaks observed were due to light scattering artefacts.

**Table 1:** Extinction coefficients determined or employed.

Species	pH	Wavelengths(nm)	E(mM^− 1^ cm^− 1^)
O (absolute spectrum)	8.75	423–460	133.3
		598–630	13.75
		825–(750 + 900)/2	0.74
Cu_A_ red minus oxidised	7.4	825–(750 + 900)/2	0.60
F minus O	8.75	579–630	(5.5)*
		606–630	1.05
		436–412	54.3
P minus O	8.75	606–630	(11.0)*
		579–630	2.15
		437–412	49.8
CO reduced minus reduced	8.75	590–606	9.2
Haem *a*_3_ & CuB reduced minus oxidised	8.75	605–630	3.5
Haem *a* reduced minus oxidised	8.75	605–630	23.5

Determined in this paper apart from: * Morgan and Wikstrom [40].

### 2.6 Spectral deconvolutions

The separated visible and NIR spectra for haem *a*, haem *a*_3_ and Cu_A_ were calculated via deconvolution of spectra in a variety of redox and ligand states. Table 2 summarises how these were calculated.

**Table 2:** Protocols for obtaining cytochrome oxidase difference spectra.

Species measured experimentally		Difference spectra calculated		Resultant species discussed in paper	Location in paper
I	*a*^2 +^*a*_3_^2 +^	I − IX	*a*^2 +^*a*_3_^2 +^ − *a*^3 +^*a*_3_^3 +^	*a*^2 +^*a*_3_^2 +^–*a*^3 +^*a*_3_^3 +^ (*dithionite*)	Fig. 2A a
		I − III	*a*^2 +^*a*_3_^2 +^ − *a*^2 +^*a*_3_^3 +^	*a*_3_^2 +^–*a*_3_^3 +^ (*dithionite*)^a^	a
		I − IV + VIII − IX	*a*^2 +^*a*_3_^2 +^ − *a*^3 +^*a*_3_^3 + ^+ *a*^3 +^*a*_3_^3 +^form − *a*^2 +^*a*_3_^3 +^ form.	*a*_3_^2 +^–*a*_3_^3 +^ (*formate*)	Fig. 6
		I − II + V − IX	*a*^2 +^*a*_3_^2 +^ + *a*^3 +^*a*_3_^2 +^ CO − *a*^2 +^*a*_3_^2 +^CO − *a*^3 +^*a*_3_^3 +^	*a*_3_^2 +^–*a*_3_^3 +^ (*CO*)	Figs. 2B, 6
II	*a*^2 +^*a*_3_^2 +^ CO	II − IX	*a*^2 +^*a*_3_^2 +^CO − *a*^3 +^*a*_3_^3 +^	*a*^2 +^*a*_3_^2 +^CO–*a*^3 +^*a*_3_^3 +^	Fig. 2A
		II − I	*a*^2 +^*a*_3_^2 +^CO − *a*^2 +^*a*_3_^2 +^	*a*_3_^2 +^CO–*a*_3_^2 +^	Fig. 2B
III	*a*^2 +^*a*_3_^3 +^	III − IX	*a*^2 +^*a*_3_^3 +^ − *a*^3 +^*a*_3_^3 +^	*a*^2 +^–*a*^3 +^ (*dithionite*)	a
IV	*a*^2 +^*a*_3_^3 +^ formate	IV − VIII	*a*^2 +^*a*_3_^3 +^form − *a*^3 +^*a*_3_^3 +^form.	*a*^2 +^–*a*^3 +^ (*formate*)	Fig. 3A
V	*a*^3 +^*a*_3_^2 +^ CO	V − IX	*a*^3 +^*a*_3_^2 +^CO − *a*^3 +^*a*_3_^3 +^	*a*_3_^2 +^CO–*a*_3_^3 +^	Fig. 2B
		II − V	*a*^2 +^*a*_3_^2 +^CO − *a*^3 +^*a*_3_^2 +^CO	*a*^2 +^–*a*^3 +^(*CO*)	Fig. 2A
VI	*a*^3 +^*a*_3_^3 +^P	VI − IX	*a*^3 +^*a*_3_^3 +^P − *a*^3 +^*a*_3_^3 +^	*a*_3_^3 +^P–*a*_3_^3 +^^c^	Fig. 4B
VII	*a*^3 +^*a*_3_^3 +^F	VII − IX	*a*^3 +^*a*_3_^3 +^F − *a*^3 +^*a*_3_^3 +^	*a*_3_^3 +^F–*a*_3_^3 +^^c^	Fig. 4B
VIII	*a*^3 +^*a*_3_^3 +^ formate	VIII − IX	*a*^3 +^*a*_3_^3 +^ form − *a*^3 +^*a*_3_^3 +^	*a*_3_^3 +^formate–*a*_3_^3 +^	b
IX	*a*^3 +^*a*_3_^3 +^ pulsed				Fig. 1

Deconvoluted difference spectra for cytochromes *a* & *a*_3_ using four methods (dithionite kinetics, CO mixed valence spectrum, CN mixed valence spectrum and formate mixed valence spectrum). Note in all cases *a* includes Cu_A_ and *a*_3_ includes Cu_B_. Reagents in italics e.g. (*formate*) in column three are used to indicate the different methods by which essentially similar liganded states were calculated. The fourth column indicates where the spectra are illustrated in this paper or where in the literature they were measured.

a See Nicholls & Wrigglesworth [20].

b See Nicholls [41].

c Compounds P and F are detectable only in the fully oxidised state.

## 3 Results

**Fig. 2:**
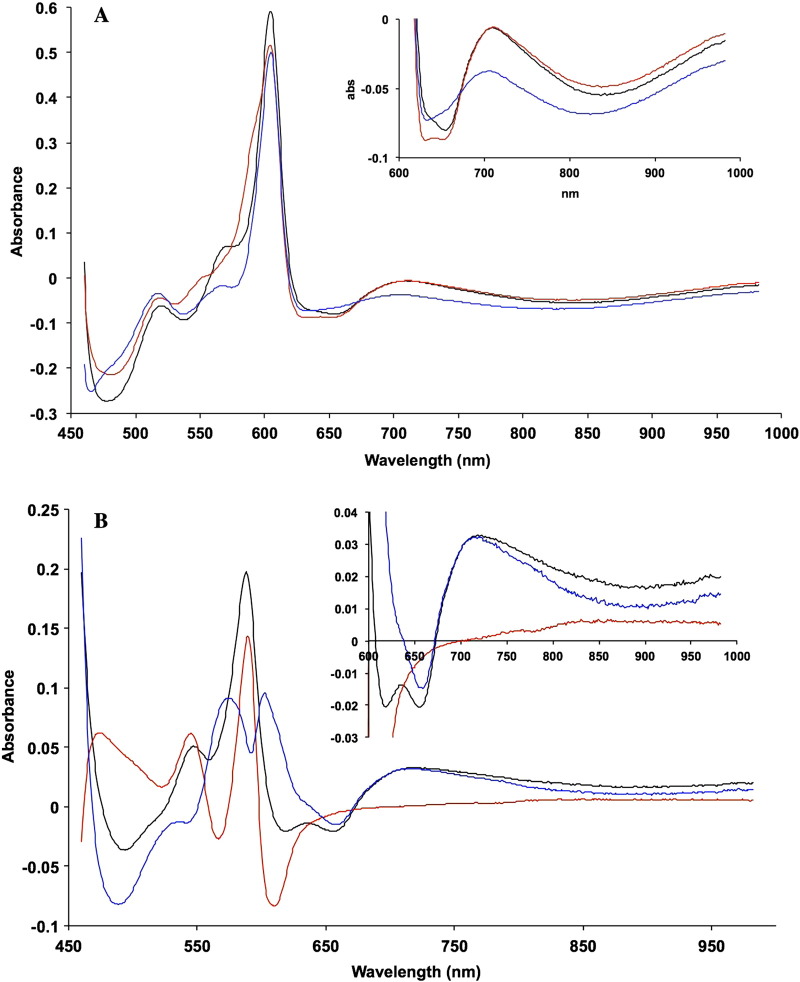
Difference spectra of the reduced, CO-liganded reduced, and mixed valence enzyme. A. Difference spectra plus/minus CO (all with haem *a* and Cu_A_ reduced): Reference spectrum was oxidised enzyme in each case (see Fig. 1). Reaction conditions as in Fig. 1. CO complexes (fully reduced and mixed valence) formed as described in Materials and methods (0.1 M Na borate buffer pH 8.75 0.1% lauryl maltoside at 22 °C). -------- fully reduced *minus* oxidised (I − IX, Table 2); fully reduced (plus CO) minus oxidised (II − IX, Table 2); & fully reduced (plus CO) minus mixed valence CO (plus ferricyanide, 20 mM) (II − V, Table 2) (i.e. haem *a* & Cu_A_ reduced minus oxidised). B. Difference spectra plus/minus CO (all with haem *a* and Cu_A_ contributions subtracted): Mixed valence CO complex was prepared as in Materials and methods. Each spectrum represents the calculated difference spectrum of the binuclear centre either in the presence or absence of CO. -------- mixed valence reduced (plus CO) minus oxidised (V − IX, Table 2); fully reduced (plus CO) minus fully reduced (unliganded) (II − I, Table 2); & mixed valence reduced minus oxidised (fully reduced minus reduced CO plus mixed valence reduced (plus CO) minus oxidised) (I − II + V − IX, Table 2).

Fig. 2 shows the visible and NIR difference spectra (reduced minus oxidised) of the oxidase obtained in the presence and absence of the binuclear centre ligand CO. In each case the reference spectrum was the oxidised enzyme (as in Fig. 1). Fig. 2A shows three types of difference spectra for the enzyme, in each of which the haem *a* and Cu_A_ are both reduced (fully reduced minus oxidised, fully reduced enzyme plus CO minus oxidised and fully reduced CO enzyme minus mixed valence CO enzyme). The inset shows the NIR spectra on an expanded scale. The Cu_A_ trough at 835 nm is similar in all three cases but the 655 nm feature (trough) shows characteristic differences. The CO-bound fully reduced enzyme shows a double trough in this region where the unliganded fully reduced enzyme and the third spectrum show only a single trough, albeit in different positions. There is also a trough in the 450–500 nm region with analogous characteristic differences. This is discussed below. Fig. 2B shows three types of difference spectra, in each of which the haem *a* and the Cu_A_ are both oxidised (mixed valence CO minus oxidised, fully reduced CO minus fully reduced, and derived mixed valence reduced minus oxidised). These therefore all provide relevant difference spectra for different states of the binuclear centre. As in Fig. 2A, the inset in Fig. 2B shows the NIR spectra on an expanded scale. The reduced minus oxidised difference spectra, both with and without CO, show a broad feature centred at 710 nm which gives rise to an anomalous apparent trough in the NIR centred near 900 nm (note that Cu_A_ is either fully reduced or fully oxidised in each of these spectral pairs). No such feature appears in the reduced CO minus reduced spectrum, whose NIR signature is essentially flat.

**Fig. 3:**
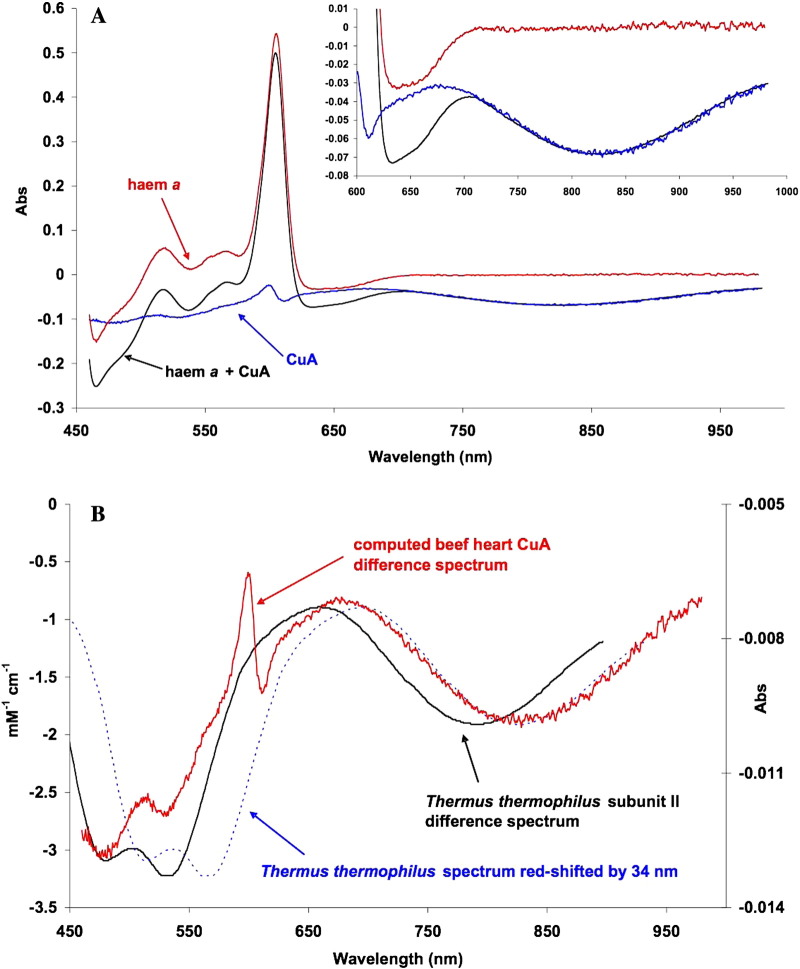
Separated redox difference spectra of cytochrome *a* and Cu_A_. 0.1 M sodium phosphate buffer pH 7.4 0.1% lauryl maltoside 22 °C. with 7.5 mM sodium formate present in the mixed valence sample (haem *a* Cu_A_ reduced/haem *a*_3_ CuB oxidised). Formate complexes formed as described in Materials and methods. A. Separated difference spectra +/− formate (haem *a* and Cu_A_): ------ formate mixed valence minus oxidised formate (haem *a* Fe^2 +^ plus Cu_A_^+^) (IV− VIII, Table 2); formate mixed valence minus calculated Cu_A_ (haem *a* Fe^2 +^); (i.e. haem *a* reduced minus oxidised). formate mixed valence minus calculated haem *a* (Cu_A_^+^) (i.e. Cu_A_ reduced minus oxidised). B. Bacterial and mammalian Cu_A_ reduced minus oxidised difference spectra. Cu_A_ difference spectrum obtained by reduction in the presence of formate (converted to extinction coefficients); -------- *Thermus thermophilus* subunit II difference spectrum (digitised version from the cytochrome oxidase Web site http://www-bioc.rice.edu/~graham/CcO.Spectra.pc). The troughs in the visible spectra are closely matched but in the NIR the bacterial subunit II spectrum is blue shifted by ~ 34 nm relative to the computed cytochrome *c* oxidase Cu_A_ . The 599 to 611 nm spike is due to the haem *a* spectral shift that occurs upon Cu_A_ reduction (see Discussion).

Fig. 3A shows some analogous spectra for the formate-inhibited system. Here the binuclear centre is held in the fully oxidised state (both haem *a*_3_ and Cu_B_) by formate [41]. Slow reduction of the resulting inhibited complex by ascorbate and a low level of TMPD permit a distinction of the Cu_A_ and haem *a* spectra because their redox potentials differ and they remain in thermodynamic equilibrium during the reduction time course. The spectra shown are the combined spectra for the reduction of both components (cf. the corresponding CO-inhibited spectra in Fig. 2A) and the deconvoluted spectra for the two separate species, haem *a* alone and Cu_A_ alone. The inset again shows the NIR spectra on an expanded scale. The isolated haem *a* spectrum is featureless in the NIR and contributes nothing to the 835 nm absorbance changes.

Only the spectra with Cu_A_ oxidised minus reduced difference components show an 835 nm band. Conversely only the haem *a* component shows a reduced minus oxidised trough in the 630–650 nm region. However, as well as affecting each other's redox potentials [42], the two centres interact spectrally: the reduced minus oxidised Cu_A_ spectrum shows a blue shift of the alpha (605 nm) peak of haem *a*. That this is a feature of the intact enzyme rather than of the Cu_A_ centre alone is shown by a comparison of the derived Cu_A_ spectrum in Fig. 3A with the available spectra of isolated prokaryotic Cu_A_ domains (subunit II species). Fig. 3B compares the beef heart Cu_A_ spectrum with that reported for isolated subunit II (Cu_A_-containing) from *Thermus thermophilus* [43]. The beef heart Cu_A_ 835 nm band is red-shifted by 34 nm compared to the prokaryotic spectrum shown here. This red shift of the NIR mammalian centre is also seen relative to the other two prokaryotic spectra in the literature—those of *Paracoccus denitrificans* [44], [45] and of *Synechocystis* [46]. The corresponding visible region components (troughs at 470 and 520 nm) are located similarly in the eukaryotic and prokaryotic cases. However, only the difference spectrum for intact mammalian enzymes shows the extra feature associated with the haem *a* 605 nm alpha peak, indicating a blue shift in the latter associated with the Cu_A_ reduction.

**Fig. 4:**
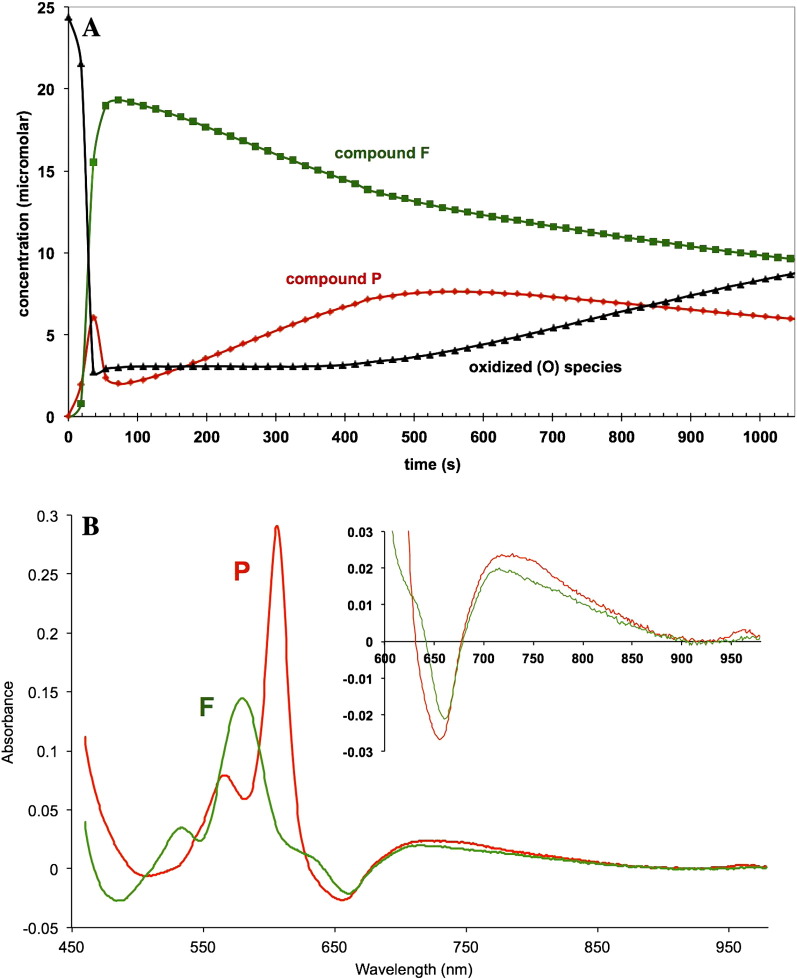
Kinetic and spectral features of the redox intermediates ‘P’ and ‘F’. A. Time course of O, F & P changes after addition of H_2_O_2_ at an alkaline pH. 24 μM cytochrome *c* oxidase in 0.1 M borate buffer pH 8.75 0.1% lauryl maltoside at 2 °C. Initial oxidation achieved by addition of 5.2 mM H_2_O_2_ measured at 10 s. ------ ‘O’ (oxidised cytochrome *c* oxidase); ‘F’ (compound F); ‘P’ (compound P). The green and red traces show the amounts of F and P formed after correcting for the contribution of the other species at the same wavelength pair (as described in Materials and methods; cf. Table 1). The concentration of ‘O’ (residual ferric enzyme) is calculated at each time point as [O] = [O]_initial_ minus ([P] + [F]). B. Difference spectra of compounds P and F (minus O). pH 8.75; other conditions as in Fig. 4A. compound F (VII − IX, Table 1); compound P (VI − IX, Table 1). Difference spectrum of P made by gassing sample with CO (see Materials and methods). Difference spectrum of F made from the peroxide-induced spectra (Fig. 4A), corrected for the contribution of species P and total occupancy.

The NIR characteristics of the enzyme with the binuclear centre in the reduced state, formate-ligated, and CO-ligated, may be compared with the corresponding oxygen intermediates, the so-called ‘P’ and ‘F’ states. Fig. 4 illustrates the results obtained. Compound P is obtained from oxidised enzyme by aerobic CO treatment [47] and compound F by H_2_O_2_ treatment [48]. The CO-induced compound P spectrum (presumed to be compound P_M_) is stable over a period of several minutes. The H_2_O_2_-induced spectra (compound F and some P species) are less stable. Fig. 4A illustrates a time course of peroxide-induced changes. On addition of peroxide to oxidised enzyme in the presence of catalase (to remove excess peroxide within a few seconds) there is immediate formation of an initial compound P species which changes to give largely compound F over a period of tens of seconds. A slow reformation of a second compound P species with the loss of the compound F is then seen over a prolonged period, which terminates with loss of both intermediates and the reformation of oxidised enzyme. The scheme (Fig. 4A & see Discussion) summarises the apparent events taking place. Fig. 4B shows the resulting difference spectra of compounds P and F at pH 8.75 (this alkaline pH maximises occupancy of the P state). In addition to the well-known 607 nm alpha peak and 560 nm ‘beta’ band, compound P shows characteristic features in the NIR region, similar to those seen for the reduced binuclear centre and the reduced-CO bound binuclear centre (Fig. 2B inset) including a trough at 655 nm and a positive feature at 710–720 nm. The difference spectrum of compound F is also shown in Fig. 4C. In addition to the recognised visible region absorbance bands at 540 nm and 580 nm there are again characteristic NIR features, the trough at 655 nm and the broad absorbance centred at 710–720 nm but extending to considerably longer wavelengths (inset).

**Fig. 5:**
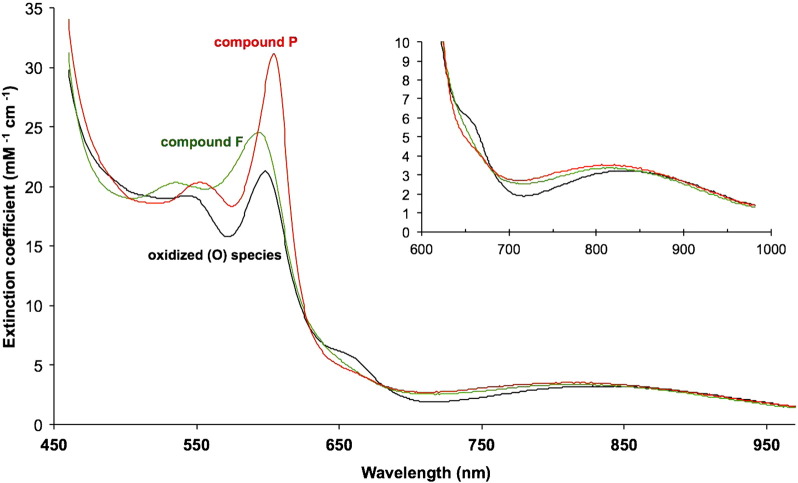
Absolute spectra of O, P, & F. Spectra are plotted in terms of millimolar extinction coefficients (cf. Table 1). ------ Black = compound O (oxidised state); Red = compound P; Green = compound F. Corrected difference spectra of F minus O and P minus O (Fig. 3B) were normalised to 100% of the starting oxidised spectrum (see Materials and methods; cf. Table 1), and then added to the original absolute oxidised spectrum obtained under the same conditions. pH 8.75, 22°°C.

From the known difference spectra and using these extinction coefficients in Table 1, the absolute spectra of the P and F species can also be obtained. Fig. 5 shows the results and the inset compares the absolute spectra for P, F & O in the NIR region. All three species show the characteristic “835 nm” band but details differ in the 650–800 nm region, because of the existence of two extra characteristic spectral features attributable to the binuclear centre:

(i) a 660 nm band in the starting oxidised species (O), presumed to be due to the ferric–cupric binuclear centre in the fully oxidised state and absent from both F and P; and,
(ii) a broad absorbance, centred at 710–720 nm but extending towards the 900 nm region, seen in both P and F forms, which distorts the 835 nm band.

**Fig. 6:**
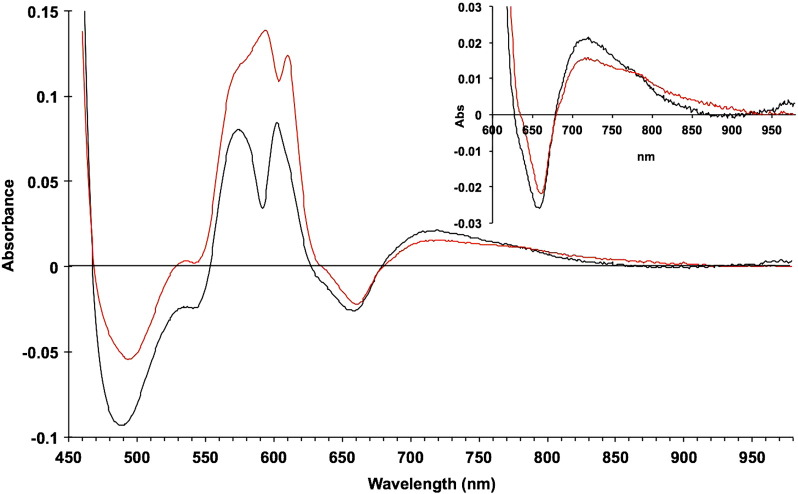
Difference spectra of the cytochrome *c* oxidase binuclear centre in the R and mixed valence R states: modulation of the ferrous spectrum by the redox state of haem *a* and Cu_A_ . ------ Black = reduced haem *a*_3_/CuB minus oxidised (from CO fully reduced and mixed valence states, I − II + V − IX, Table 2); conditions as in Fig. 2. Red = reduced haem *a*_3_/CuB minus oxidised (from formate-bound fully oxidised and mixed valence states, (I − IV + VIII − IX), Table 2); conditions as in Fig. 3.

These features are most clearly seen in appropriate difference spectra. Fig. 6 summarises and extends the observations in Fig. 4. This compares not only the P and F spectra but also the putative ferrous *a*_3_/cuprous CuB spectrum obtained in two ways. The latter can be obtained either by subtracting the haem *a*/Cu_A_ components from a system with the binuclear centre maintained reduced in presence of CO, or by subtracting the haem *a*/Cu_A_ components from a system with the binuclear centre maintained oxidised in presence of formate. Both of these derived spectra and the P & F forms have very similar NIR features involving a 660 nm trough and a 715–920 nm broad peak (compare Fig. 4, Fig. 6).

## 4 Discussion

This paper describes the first characterisation of both the visible and NIR spectra of mammalian cytochrome oxidase, quantitatively separated into components attributable to haem *a*, haem *a*_3_, Cu_A_ and the oxygen intermediates P and F. It confirms that the dominant NIR species is the Cu_A_ centre and its associated redox state changes. However, a new NIR feature has also been characterised that is associated with the binuclear centre. Although broad, this is larger than other absorption bands that have previously been reported in this spectral region. Thus the *Eschericia coli* cytochrome *bo* reduced minus oxidised difference spectrum ([22] has a 758 nm peak (~ 0.25 mM^− 1^ cm^− 1^) which becomes less intense and shifted to 745 nm upon CO addition (~ 0.05 mM^− 1^ cm^− 1^). A similar 784 nm feature is seen with the beef heart enzyme [21]. But these are much smaller than the 715–920 nm feature observed in this paper.

These broad NIR features are common to all the difference spectra involving changes in the binuclear centre, including simple reduction, reduction with ligand (CO) binding, compound P formation and compound F formation. They may thus account for some of the uncertainty concerning the relative contributions of Cu_A_ and other species to the net NIR changes centred at 835 nm. The latter are purely diagnostic of Cu_A_ reduction when the binuclear centre is held in a constant redox and ligation state during the changes being monitored. This is most likely the case both aerobically in vitro and during in vivo steady state studies. Under other conditions the situation may prove more complicated.

Use of the 835 nm absorbance must therefore take this possible contribution from the binuclear centre into account. The latter can be held in a constant form either by adding a ligand-cyanide, formate, or carbon monoxide—or by keeping the system fully aerated (which locks the binuclear centre in an oxidised steady state). Changes in the redox state of cytochrome *a*, by contrast, do not affect the 835 nm absorbance (Fig. 3A). NIR reduced minus oxidised spectra of cytochrome oxidases from *Bacillus subtilis* membranes also show that the *aa*_3_ quinol oxidase lacking the Cu_A_ centre has a featureless difference spectrum between 700 and 900 nm, whereas the *caa*_3_ oxidase has a broad NIR feature similar to that of the mitochondrial *aa*_3_ oxidase, except that the *caa*_3_ NIR feature has a lower amplitude and is blue-shifted by 40 nm compared to that of mitochondrial oxidase [14].

Some literature discrepancies may be resolvable by attention to these details. For example, by carefully ensuring a constant starting state for the enzyme, we were able to monitor the redox state of Cu_A_ at 835 nm with varying turnover in a kinetically satisfactory way [16]. This contrasted with an earlier study in which initiation of turnover secured an apparent increase in 835 nm absorbance prior to decreases characteristic of reduction [49]. In that case a consistent reduction profile for Cu_A_ could only be achieved in the presence of inhibitors (formate or azide), which lock the binuclear centre into a single spectroscopic state. The initial positive change in the “apparent” Cu_A_ state was almost certainly due to a change at the binuclear centre with a 715–920 nm absorbance increase. As with turnover studies so with redox titrations. Although several groups [50] have obtained classical n = 1 Nernst potentials for the 835 nm component, others showed that the species identified in redox titrations are mixtures in which the 835 nm component may be a minority form [51]. Anaerobic titrations involve continuous redox changes in all the redox-active centres; as several potentials are close in magnitude [16]. As the redox states of any one centre affect both redox potentials and sometimes spectra of the other centres a simple deconvolution becomes impossible.

**Fig. 7:**
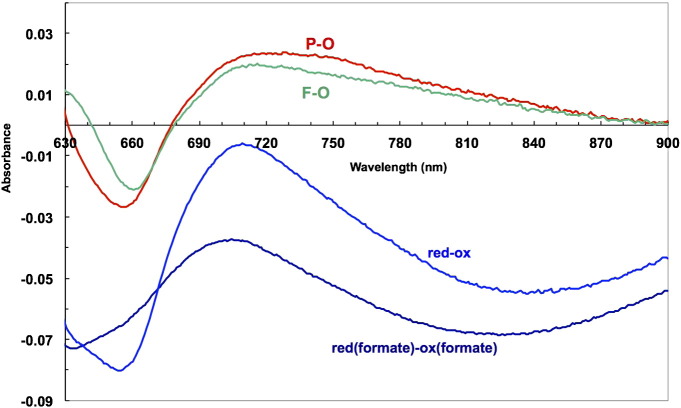
NIR difference spectra of oxidase species with and without CuA redox features. Data from Fig. 1, Fig. 3A and 4B. pH 8.75 & pH 7.4; 24.5 μM cytochrome *c* oxidase; other conditions as in Fig. 4A & B. compound F minus oxidized (O) (VII − IX, Table 2); pH 8.75. compound P minus oxidized (O) (VI − IX, Table 2); pH 8.75. fully reduced minus oxidized (O) (I − IX, Table 2); pH 8.75. mixed valence (cytochrome *a*^2 +^, CuA^+^) formate complex minus oxidised formate complex (IV − VIII, Table 2); pH 7.4.

Spectral interactions between centres are illustrated for haem *a* and Cu_A_ in Fig. 3A & B and for the reduced binuclear centre and haem *a* in Fig. 6. The separated Cu_A_ and haem *a* spectra (Fig. 3B) show an alpha peak shift within the computed Cu_A_ spectrum that must reflect a very small shift in the alpha peak of haem *a* as Cu_A_ is reduced. When the ‘resting’ enzyme is reduced by dithionite the initial (predominantly haem *a* and Cu_A_) and final (predominantly haem *a*_3_ and CuB) effects yield by kinetic deconvolution the difference spectra as shown in Nicholls & Wrigglesworth [20]. The reduced minus oxidised spectrum of the binuclear centre is characterised by a broad and rather flat-topped absorbance band from 560 to 610 nm. However when obtained by double difference methods (Table 2, Fig. 6) the systems that involve subtraction of a spectrum with haem *a* reduced and the binuclear centre reduced and liganded (CO), or oxidised and liganded (formate), show substantially different spectra in the 600 nm region. The formate system shows a small alpha peak shift, indicating a difference from the pure redox case obtained with dithionite. The carbon monoxide system shows a more substantial shift spectrum in this region. The haem a contribution in the 580–610 nm region is very dependent on the redox and ligation state of the binuclear centre, It is therefore all the more surprising to see such close similarities in the NIR region for all these difference spectra (Figs. 4B, Fig. 6, Fig. 7). The trough at 655 nm is clearly a reflection of the common changes involved when the ferric–cupric magnetic coupling disappears, both in reduction and in oxidation (to P or F states) of the binuclear centre. But the broad positive absorbance changes in the 715–920 nm region must reflect a change or changes in that centre that are common to the unliganded reduced state (formate data), the CO-bound reduced state, compound P and compound F. We do not currently have a simple model to explain this.

Fig. 7 directly compares the NIR spectra of the various species described in this paper. It illustrates the fact that, even at 100% occupancy, the 715–920 nm feature of the oxygen intermediates P and F does not interfere with methods that directly rely on the spectral shape of the Cu_A_ e.g. a three point drop between 790 and 900 nm centred at the 835 nm trough. However, there is the possibility for interference if the complete spectrum is fit over the NIR region and the additional broad absorbing feature is ignored. The NIR absorbance changes of the oxidase have been used for in vivo studies of respiration because the strong NIR signal from the enzyme offsets in part its lower concentration in tissue compared to haemoglobin or myoglobin. However the effects seen in the presence of inhibitors reacting either with the reduced state (e.g. CO) or the oxidised state (e.g. cyanide and formate) show a need for caution in attempting quantitative interpretations.

We conclude that, although the primary contributor to the cytochrome oxidase near infrared spectral signature is cupric Cu_A_, large changes in the population of the oxygen intermediates, P and F, have the potential to confuse the interpretation of in vivo data. Although our in vitro studies on the isolated enzyme suggest that P and F are generally minority populations during steady state turnover [16], there are alternative views [52], most recently from mitochondrial studies [53]. Therefore, although our results confirm the usefulness of NIR to monitor oxidase activity in vivo, we suggest appropriate caution about data interpretation.

## Funding

This work was supported by a grant to CEC from the UK Biotechnology and Biological Sciences Research Council [BB/D017858/1]. The funder played no role in any of: study design; the collection, analysis and interpretation of data; the writing of the report; and the decision to submit the article for publication.
